# Supplementary material for: A Dual Role of Osteopontin in Modifying B Cell Responses
Source: Biomedicines. 2023 Jul 12;11(7):1969. doi: 10.3390/biomedicines11071969 (PMC10377065; doi:10.3390/biomedicines11071969)
Supplement: Supplementary file 1 [file biomedicines-11-01969-s001.zip › biomedicines-2445127-supplementary.pdf]

Supplementary material

for

A dual role of osteopontin in modifying B cell responses

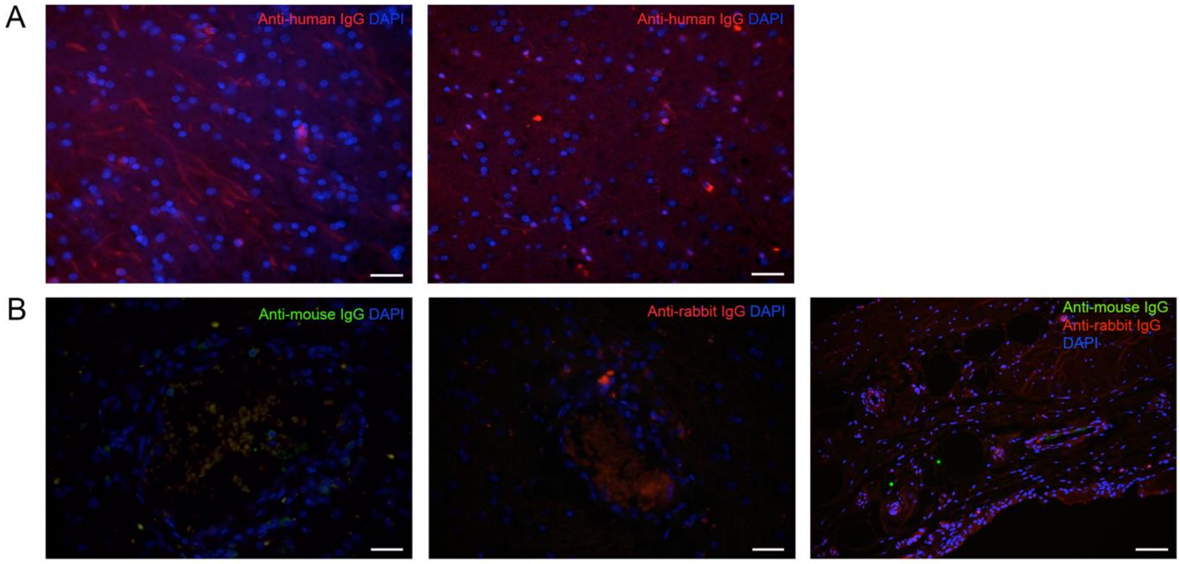

Figure S1. Control stainings for immunohistochemistry. (**A**) Representative images of *n* = 2 MS brain tissue sections stained with polyclonal rabbit anti-human IgG and a Cy3-coupled anti-rabbit secondary antibody. Scale bars represent 50 µm. (**B**) Brain tissue from an MS patient with CD20$^+$ B cell aggregates stained with Cy2- (left panel) or Cy3-coupled (middle panel) secondary antibody only, respectively, corresponding to the CD20 and OPN staining. Scale bars represent 50 µm. Synovial tissue from a CD20$^+$ B cell aggregate-positive RA patient stained with both Cy2- and Cy3-coupled secondary antibodies (right panel) corresponding to the CD20 and OPN staining . The scale bar represents 100 µm. MS = multiple sclerosis; OPN = osteopontin; RA = rheumatoid arthritis.

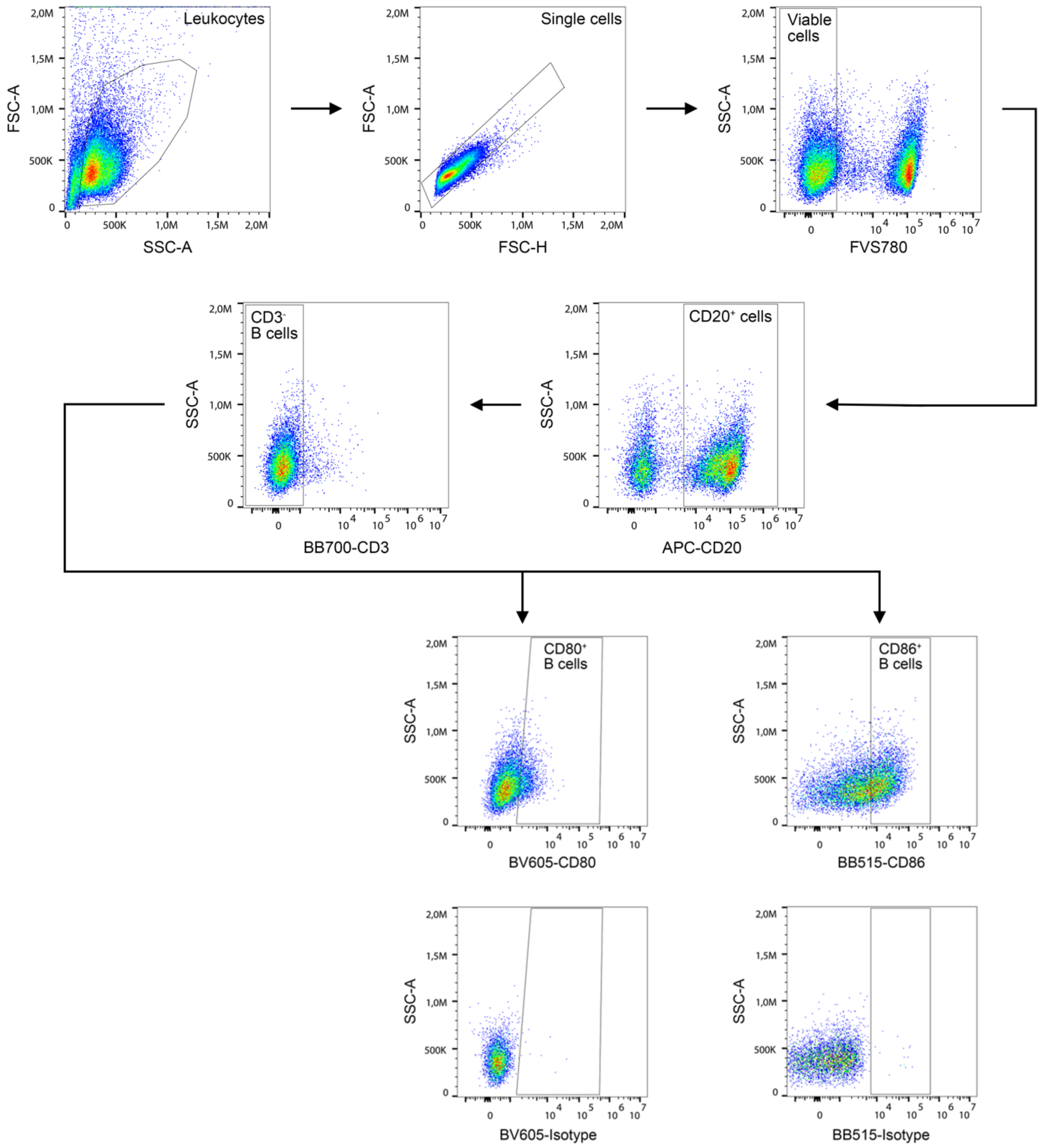

Figure S2. Representation of the gating strategy for B cell activation [1]. A leukocyte gate was set and single cells were determined by forward scatter (FSC)-height (FSC-H) and FSC-area (FSC-A). Dead cells were excluded, following which $CD20^+$ cells were identified and $CD20^+CD3^-$ B cells were discriminated from the $CD20^+CD3^+$ cell population. Finally, the $CD20^+CD3^-CD80^+$ and $CD20^+CD3^-CD86^+$ B cell populations were identified. Isotype controls (for CD80 and CD86 staining) were included for the gating strategy to identify the $CD80^+$ and $CD86^+$ B cell population.

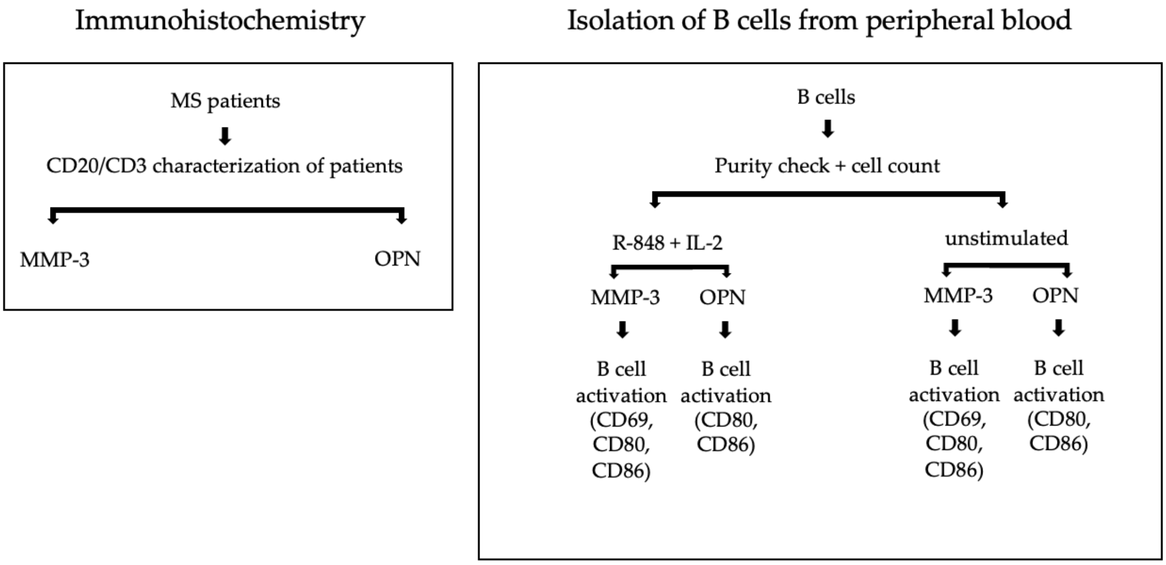

Figure S3. Materials and methods with an overlap between [1] and the current manuscript. MMP-3 = matrix metalloproteinase-3; OPN = osteopontin; R-848 = synthetic toll-like receptor 7/8 agonist.

	**A**	**B**	**C**	**D**	**E**	**F**	**G**	**H**
**1**	Pos	Pos	Neg	Neg	GCSF	GM-CSF	GRO (α, β, γ)	GRO-α
**2**	Pos	Pos	Neg	Neg	GCSF	GM-CSF	GRO (α, β, γ)	GRO-α
**3**	IL-1α	IL-2	IL-3	IL-5	IL-6	IL-7	IL-8	IL-10
**4**	IL-1α	IL-2	IL-3	IL-5	IL-6	IL-7	IL-8	IL-10
**5**	IL-13	IL-15	IFN-γ	MCP-1	MCP-2	MCP-3	MIG	RANTES
**6**	IL-13	IL-15	IFN-γ	MCP-1	MCP-2	MCP-3	MIG	RANTES
**7**	TGF-β1	TNF-α	TNF-β	BLANK	BLANK	BLANK	BLANK	Pos
**8**	TGF-β1	TNF-α	TNF-β	BLANK	BLANK	BLANK	BLANK	Pos

Figure S4. Layout of the antibody array used for investigating cytokines. GCSF = granulocyte colony-stimulating factor; GM-CSF = granulocyte-macrophage colony-stimulating factor; GRO = growth-regulated proteins; IL = interleukin; IFN = interferon; MCP = monocyte chemoattractant protein; MIG = monokine induced by gamma interferon; Pos = positive; Neg = negative; RANTES = regulated on activation, normal T cell expressed and secreted (also known as CCL5); TNF = tumor necrosis factor; TGF = transforming growth factor.

Table S1. Purity of B cells isolated from the different donors and their use in the different assays.

	**Purity in %** **($CD45^+CD19^+$)**	**B cell activation panel** **(CD80, CD86)**		**ELISA** **(IL-6/IL-10)**
**Donors**		**Unstimulated**	**Stimulated with R-848 + IL-2**	**Supernatant from stimulated cells**
**1**	87.8		x	x
**2**	82.3*	x		
**3**	94.6*	x	x	x
**4**	87.3	x	x	x
**5**	91.7		x	x
**6**	90.1		x	x
**7**	97.9*	x		
**8**	91.9*	x		
**9**	93.7		x	x
**10**	90.8	x		

* = B cells of donors used for both the current study and [1]; IL = interleukin; R-848 = synthetic toll-like receptor 7/8 agonist.

Table S2. Integrated densities of the dot blot array.

Relative expression to Ctrl [Sample/Ctrl]	Name of cytokine
Not measurable	GCSF
	GM-CSF
	GRO
	GRO-α
	IL-1a
	IL-2
	IL-3
	IL-5
0.888	IL-6
0.075	IL-7
2.716	IL-8
1.100	IL-10
0.870	IL-13
0.527	IL-15
0.155	IFN-γ
0.762	MCP-1
Not measurable	MCP-2
	MCP-3
	MIG
2.497	RANTES
0.895	TGF-β1
0.903	TNF-α
1.290	TNF-β

Ctrl = B cells without rOPN treatment; GCSF = granulocyte colony-stimulating factor; GM-CSF = granulocyte-macrophage colony-stimulating factor; GRO = growth- regulated proteins; IL = interleukin; IFN = interferon; MCP = monocyte chemoattractant protein; MIG = monokine induced by gamma interferon; Pos = positive; Neg = negative; rOPN = recombinant osteopontin; RANTES = regulated on activation, normal T cell expressed and secreted (also known as CCL5); Sample = B cells from the same donor with rOPN treatment; TNF = tumor necrosis factor; TGF = transforming growth factor.

Supplementary references

1. Chunder, R.; Schropp, V.; Jabari, S.; Marzin, M.; Amor, S.; Kuerten, S. Identification of a novel role for matrix metalloproteinase-3 in the modulation of B cell responses in multiple sclerosis. *Front. Immunol.* **2022**, *13*, 1025377. https://doi.org/10.3389/fimmu.2022.1025377.
